# Supplementary material for: Reduced Humoral and Cellular Immune Response to Primary COVID-19 mRNA Vaccination in Kidney Transplanted Children Aged 5–11 Years
Source: Viruses. 2023 Jul 14;15(7):1553. doi: 10.3390/v15071553 (PMC10384144; doi:10.3390/v15071553)
Supplement: Supplementary file 1 [file viruses-15-01553-s001.zip › viruses-2480664-supplementary.pdf]

Fluorochrome	Antigen	Clone	Supplier
BV510	CD45RA	HI100	Biolegend
BV605	CD4	SK-3	Biolegend
BV650	CD27	O323	Biolegend
BV785	CD3	OKT3_	Biolegend
AF488 / FITC	CD69	FN50	Biolegend
PE-Cy7	CD134 (OX40)	Ber-ACT35	Biolegend
APC	CD137	4B4-1	Biolegend
AF700	CD8	RPA-T8	Biolegend
APC-Cy7	LiveDead DUMP	NIR	Life

Supplementary Table S1: Flow cytometry reagent list.

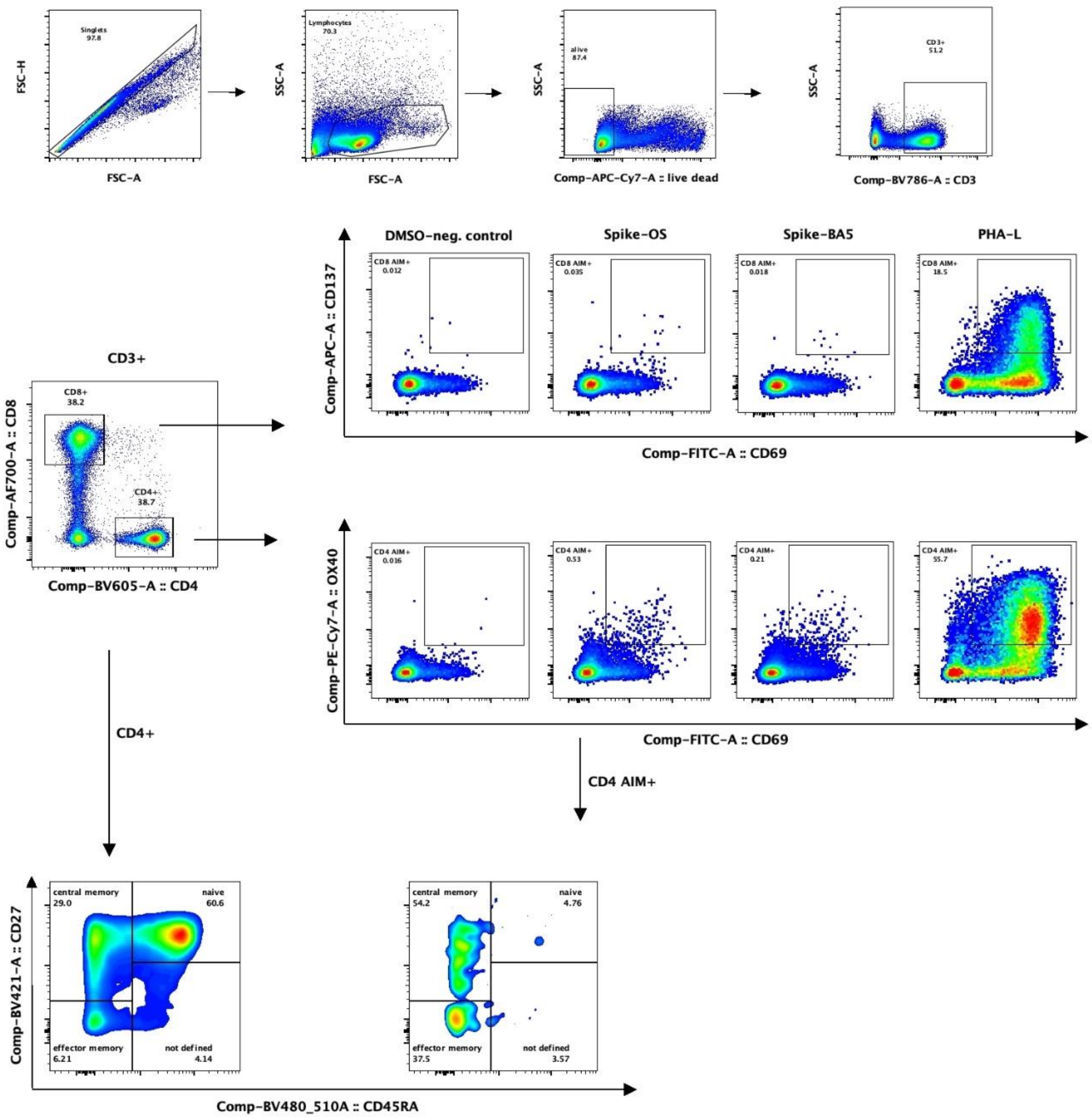

Supplementary Figure S1: Manual gating strategy for lineage and activation-induced markers.

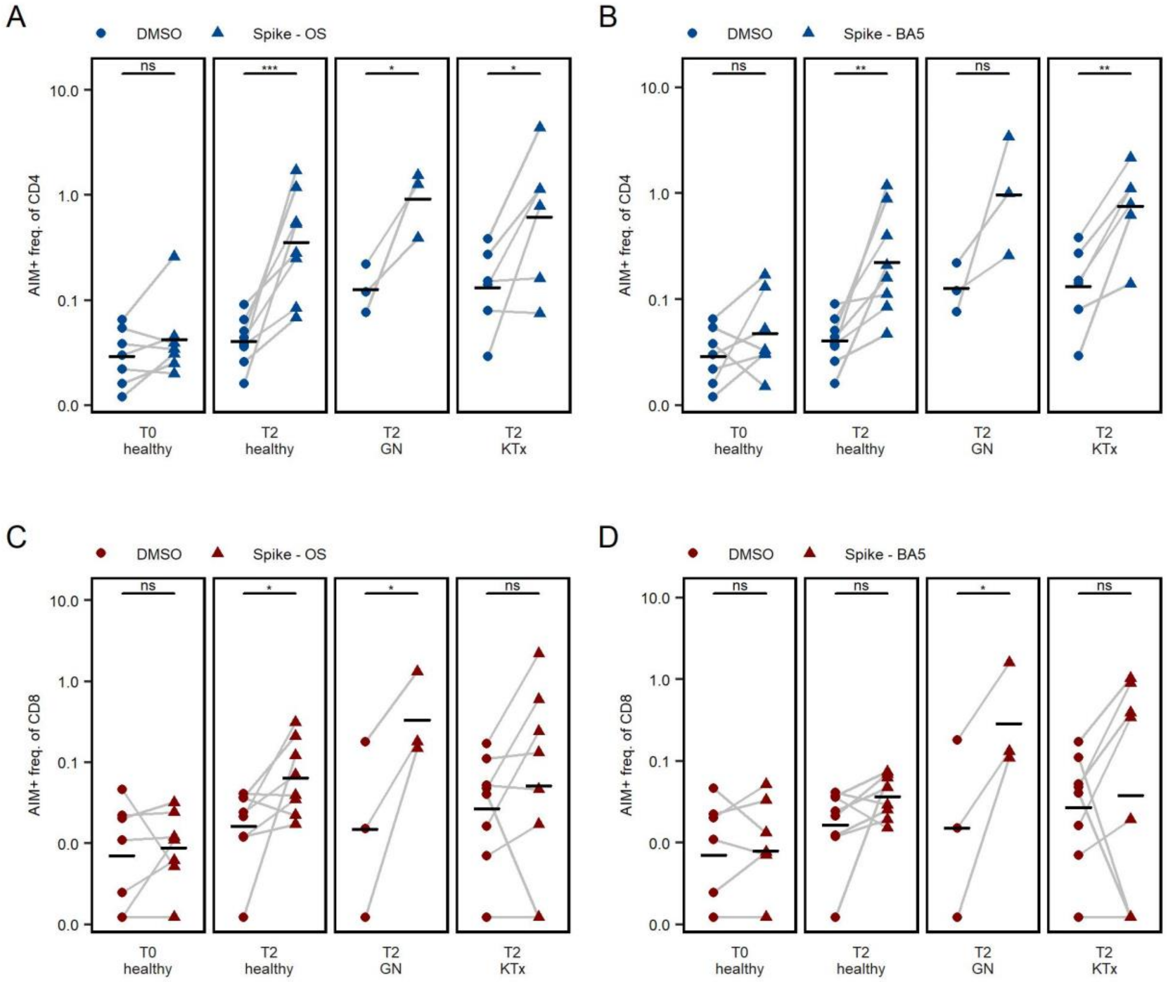

Supplementary Figure S2: Comparison between the frequency of AIM+ cells following stimulation with Spike-OS and Spike-BA.5 and the frequency of AIM+ cells following DMSO exposure based on the expression of CD69 and OX40 in CD4+ (A + B) as well as CD69 and CD137 in CD8+ (C + D). Horizontal lines are used to represent mean values. P values were determined using paired, one-sided t tests.

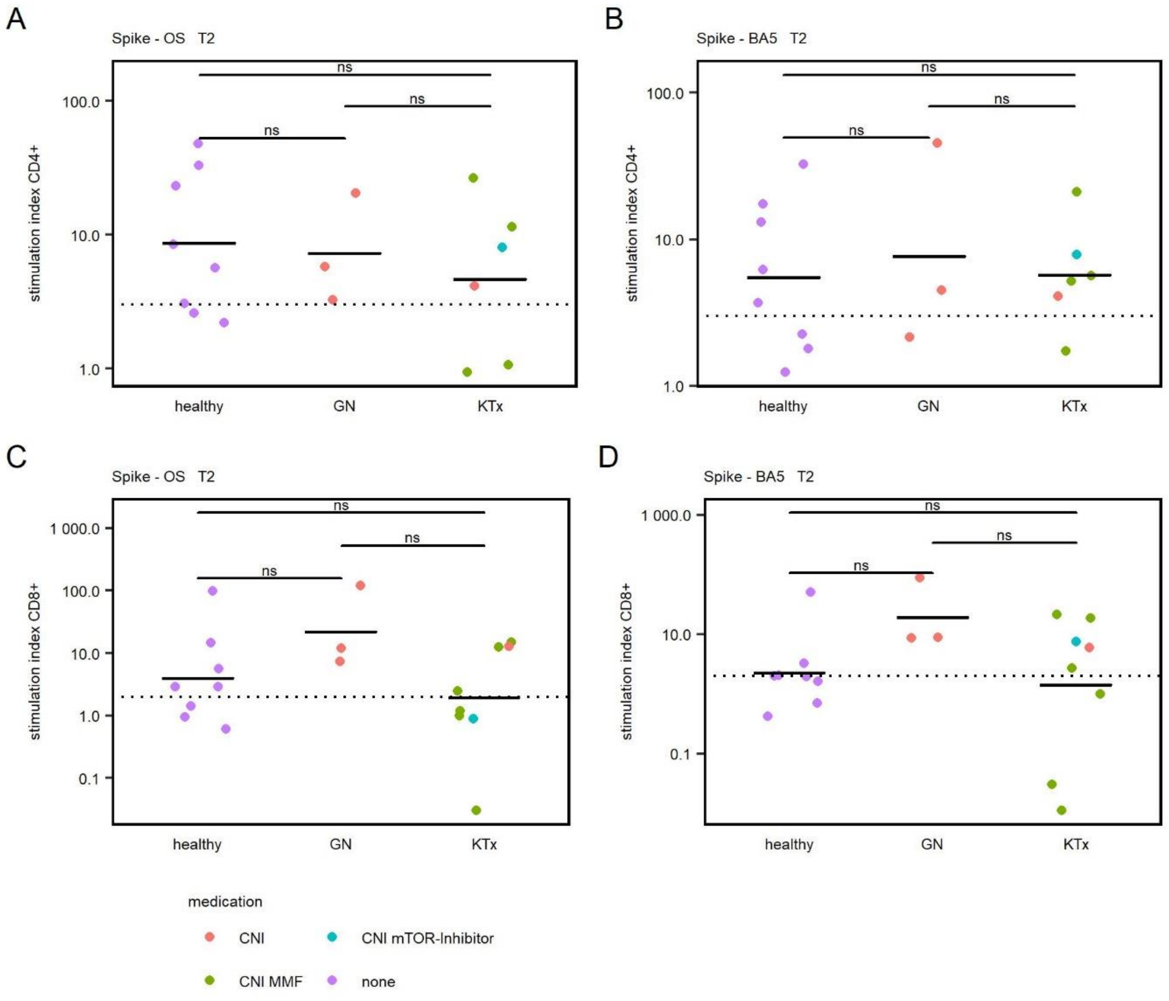

Supplementary Figure S3: T cell responses to peptide stimulation displayed as Stimulation Index. Dashed vertical line indicates threshold to be defined as response. Different medications are marked as different colors. Mean values are represented by horizontal lines. P values were calculated using One-way ANOVA and post hoc pairwise t-tests.
CNI: calcineurin-inhibitors, MMF: mycophenolate mofetil
